# Head movement compensation in real-time magnetoencephalographic recordings

**DOI:** 10.1016/j.mex.2014.10.008

**Published:** 2014-11-03

**Authors:** Graham Little, Shaun Boe, Timothy Bardouille

**Affiliations:** a Biomedical Translational Imaging Centre (BIOTIC), IWK Health Sciences Centre, Halifax, Nova Scotia, Canada; b Department of Diagnostic Imaging, IWK Health Sciences Centre, Halifax, Nova Scotia, Canada; c Laboratory for Brain Recovery and Function, School of Physiotherapy, Dalhousie University, Halifax, Nova Scotia, Canada; d Department of Computer Sciences, Dalhousie University, Halifax, Nova Scotia, Canada

**Keywords:** Brain computer interface (BCI), Magnetoencephalography (MEG), Neurofeedback, Real-time analysis

## Abstract

Neurofeedback- and brain-computer interface (BCI)-based interventions can be implemented using real-time analysis of magnetoencephalographic (MEG) recordings. Head movement during MEG recordings, however, can lead to inaccurate estimates of brain activity, reducing the efficacy of the intervention. Most real-time applications in MEG have utilized analyses that do not correct for head movement. Effective means of correcting for head movement are needed to optimize the use of MEG in such applications. Here we provide preliminary validation of a novel analysis technique, real-time source estimation (rtSE), that measures head movement and generates corrected current source time course estimates in real-time. rtSE was applied while recording a calibrated phantom to determine phantom position localization accuracy and source amplitude estimation accuracy under stationary and moving conditions. Results were compared to off-line analysis methods to assess validity of the rtSE technique. The rtSE method allowed for accurate estimation of current source activity at the source-level in real-time, and accounted for movement of the source due to changes in phantom position. The rtSE technique requires modifications and specialized analysis of the following MEG work flow steps.

• Data acquisition
• Head position estimation
• Source localization
• Real-time source estimation

This work explains the technical details and validates each of these steps.

## Method details

### Data acquisition

The calibrated phantom (ElektaOy, Finland) contains 32 current sources of known position and orientation and four HPI coils inside a hemisphere casing. Sinusoidal currents tangential to the hemisphere casing can be driven at each source location independently to generate an alternating magnetic field on the MEG sensors. Eight superficial current sources (i.e., at a distance of 64 mm from the phantom origin) were selected for use in this study. The selected current sources allowed for maximum spatial disparity between sources, to investigate activation across the sensor array. These sources can be localized with millimeter accuracy based on the MEG data using a current dipole model [1]. The HPI coils on the phantom are wire loops that can be activated to generate a magnetic field source that is easily localized by the MEG sensor array. Before the MEG acquisition, the positions of the HPI coils were digitized using the FASTRAK digitization system (Polhemus, USA). The phantom was then secured in a stationary position under the MEG helmet with the capacity to manually adjust the position of the phantom during the scan to simulate head movement.

All data was acquired on a MEG system containing 204 planar gradiometers and 102 magnetometers (ElektaOy, Finland). The MEG system is housed in a lightly shielded room, which utilized active shielding to eliminate environmental magnetic interference (MaxShield; ElektaOy, Finland). Data were sampled at 1000 Hz with a bandwidth of 0.10–330 Hz, and recorded to a file for off-line analysis. Event markers indicating the onset of phantom current source activation were recorded concurrently.

Simultaneous with the off-line file recording, one-second MEG data segments were transferred to a real-time analysis computer for immediate analysis. Real-time analysis of MEG data was performed using the Matlab software environment (version 7.10.0.499 R2010a, Mathworks, USA) installed on an HP Desktop Computer, running Ubuntu version 12.04. The software for real-time data transfer was provided by the Fieldtrip toolbox [2]. This open source toolbox (version 20110727) was installed on both the Elekta MEG acquisition workstation, and the real-time analysis computer. The technical aspects of the real-time data transfer server and client, including selection of variable data segment lengths, have been described previously [3].

Only the 204 planar gradiometers were analyzed in real-time. The magnetometers were active during acquisition to facilitate active shielding. Magnetometers were not included in the real-time analysis for two reasons. Firstly, the magnetometers are more susceptible to environmental interference than the planar gradiometers. This interference can be attenuated via signal space separation (SSS) [4]. However, the SSS algorithm was not implemented in the real-time analysis described in this paper due to the significant processing power requirements. Secondly, the SSS algorithm also attenuates distortions of the measured magnetic fields due to the active shielding. This distortion is essentially isotropic across any given planar gradiometer sensor, and thus has no net effect on the measured signal. As such, active shielding mainly distorts the magnetic field data at magnetometer sensors and the effect is negligible on planar gradiometers.

At the start of the study, a short (10 s) “localizer” scan was recorded with the phantom in a stationary position. The HPI coils were activated continuously during the collection at unique driving frequencies between 293 and 321 Hz. No current sources were activated during this scan. This localizer dataset was passed to the real-time analysis computer to establish an initial high-resolution HPI coil localization. Specifically, the HPI localization data was read from the localizer data file using custom software created in the C programming language with a vendor supplied C interface which implements basic file input–output operations on Elekta formatted data files. Following this, Experiment 1 tested the accuracy of the real-time analysis for HPI coil localization. MEG data were collected for 326 s with the phantom in a stationary position, all HPI coils active, and no current source active. Experiment 2 tested the accuracy of the real-time analysis for current source localization. The phantom remained in a stationary position with all HPI coils active. During this scan, each of the eight superficial current sources was consecutively activated with 2 cycles of a 20 Hz sinusoid every 350 ms at a magnitude of 1000 nAm for 100 s. Experiment 3 tested the accuracy of HPI coil localization and source estimation during real-time analysis in the movement condition. All HPI coils and a single current source were activated. In this scan, after 10 s of baseline data were collected, a technician inside the magnetically shielded room moved the phantom once every 10–20 s to approximate sustained head translations and rotations of several centimeters and degrees, respectively. The speed and magnitude of the movements were on a similar scale to those reported in challenging human cases [5]. Also, the largest possible current source magnitude was used to provide a maximal signal-to-noise ratio at the MEG sensors for measuring small changes in estimated source strength induced by movement.

### Data analysis – head position estimation

Head position estimation occurred in the localizer scan and all three experiments. For each one-second data segment passed to the real-time computer, we calculated the magnetic field strength generated at each sensor due to the activation of each HPI coil. First, a cross-talk correction matrix was applied to the MEG data to eliminate cross-talk interference between sensors [6]. For each sensor, the data were then baseline corrected based on the mean amplitude over the data segment. Following this, a continuous signal decomposition was performed at each coil frequency to determine the sine (real) and cosine (imaginary) component of the decomposition of the magnetic field due to each HPI coil [7]. At each coil frequency, the two components for all sensors were plotted to a complex plane, and a linear regression of these data was performed. Real values indicating magnetic field strength at the coil frequency were determined by projecting the components for each sensor to the linear regression. For each HPI coil, the resultant estimates of magnetic field strength across the sensors defined the “measured” MEG field data.

Using the last one-second data segment collected in the localizer scan, the rigorous fitting method described below was applied to each HPI coil to provide accurate estimation of coil position, orientation and magnitude. The resulting parameters for each coil defined a rigid body, with the relative positions, orientations and magnitudes of all HPI coils fixed. For the rigorous fitting method, an unconstrained multivariable optimization algorithm (Nelder–Mead simplex direction search) [8] was applied to the final one-second data segment to estimate six parameters (*x*, *y*, *z*, location; *x*, *y*, *z* magnitude/orientation) for each HPI coil. The initial parameters for the optimization were provided by the acquisition software's initial head position estimation. Based on these values, the magnetic field generated orthogonal to each MEG sensor by an HPI coil modeled as a magnetic dipole defined the “calculated” MEG field data [9]. For each coil, the algorithm iteratively perturbed the parameters to optimize the least-squares error between the “measured” and “calculated” field data. The optimization algorithm was performed three times per coil. In the first optimization, only the magnitude of the magnetic dipole was allowed to vary. In the second optimization, only dipole orientation was allowed to vary. In the final optimization, only the location was allowed to vary. This approach provided a magnetic dipole location and orientation/amplitude that remained stable with additional optimizations.

The approach described above accurately localized each HPI coil. However, this method required 12 iterative processes (three for each coil) and was, thus, not feasible for real-time analysis. Therefore, once the initial coil localizations were completed, the four coil positions and magnitude/orientations defined a rigid body. During the subsequent experiments, HPI coil localization was performed by iteratively transforming the position of the rigid body as a whole, as described below. Therefore, only one iterative process was required, which substantially reduced processing requirements. Minimizing the amount of time required for head position estimation following the localizer scan provided the additional time necessary for rtSE processing (e.g., filtering, lead-field calculation, source estimation).

For all subsequent data segments passed to the real-time computer, a single six-parameter iterative algorithm optimized the translation and rotation of the HPI coil rigid body by minimizing the least-squares error between all four “measured” and “calculated” fields in one step. To further reduce processing time, a constrained optimization algorithm (active-set) [10] was used. Additionally, the optimized rigid body transform from the previous data segment was used as an initial guess, and the rigid body transformation for each data segment was constrained to 1 cm translation and 3° rotation in each axis. This constraint provided reasonable processing speed for real-time applications, while being able to compensate for head velocities experienced in all but severe cases during neuroimaging studies [5]. To reduce the chance of spurious findings due to bad data for one coil, only the three coils that generated the lowest least-squares error were used for the error calculation. For each data segment, a coordinate frame transformation matrix was determined based on the optimized parameters and saved to a file.

At the completion of each experiment, the HPI coil positions over time were calculated using transformation matrices for each data segment. The HPI position estimation was also completed using the standard offline approach implemented in the vendor-supplied MaxFilter software package. Real-time and offline HPI coil position estimates were compared between methods over time, as described in the statistical analysis section below.

### Data analysis – real-time source localization

Phantom current sources were localized using the real-time computer in Experiment 2 and compared to the known positions. Real-time averaging for each current source was completed as described below. As each data segment was passed to the real-time analysis computer, sections of data that included an event marker indicating current source activation were isolated as “trials”. Each trial was synchronized to the event marker and included 100 ms of data before and 200 ms of data after the marker. Trials were averaged separately for each current source. As trials were added, the average data were baseline corrected to the mean across the trial, and a Hanning window and low pass filter at 40 Hz were applied to eliminate edge effects and high-frequency interference. Finally, data were baseline corrected based on the 50 ms prior to the activation onset. The running inter-trial average was displayed on the real-time analysis computer for quality assurance during collection. After 100 trials were averaged for each current source, the measured MEG field data at the peak latency of 31 ms post-onset defined the “measured” MEG field data.

For each current source, localization was accomplished using equivalent current dipole (ECD) modeling [11]. An initial guess for the ECD location was calculated using a grid-search approach. MEG field data were calculated for ECDs on a spherical grid with a radius of 8 cm and spacing of 1 cm centered on the origin of the MEG system. Three ECDs were calculated at each position – one in each cardinal axis. The least-squares error between the measured and calculated field data was used to find the best-fit ECD. Following this, an unconstrained non-linear least-squares error optimization (Broyden-Fletcher-Golfarb-Shanno) [12–15] further refined the ECD position and orientation. The initial parameters for this optimization were the location determined from the grid search, and a moment of zero magnitude. Real-time estimated locations of all eight ECDs were compared to known locations of the current sources.

### Real-time source estimation (rtSE)

In Experiment 3, 485 s of MEG data and event markers were collected while the HPI coils and one of the eight superficial current sources in the phantom were activated. Every 10–20 s, the phantom was moved by a MEG technician. One-second MEG data segments were passed to the real-time analysis computer and baseline corrected based on the mean amplitude across the whole segment. Following this, the lead-field matrix was applied to provide an on-line estimate of the time course of the current source magnitude. We used the current source position and orientation estimated in Experiment 2 to calculate the related lead-field matrix transformation.

Importantly, ECD fitting determines the current source position and magnitude/orientation in the coordinate frame of the MEG system, not in the coordinate frame of the phantom. As such, source estimation can report inaccurate values for the amplitude of the current source in the case of movement. To correct for this effect, the HPI coil positions were estimated for each data segment as the data were collected using the rigid body algorithm described above. The position and orientation of the ECD was then updated in real-time based on the rigid body transformation matrix from the same data segment. The lead-field matrix was then recalculated for the new position and orientation to provide an updated estimate of source activity. All of these processes were completed in the MATLAB environment in ∼500 ms, such that source estimation with movement correction could occur in real-time.

Following the rtSE process, a Hanning window and low-pass filter at 50 Hz were applied to the data. The estimated source data and HPI positions for each data segment were saved to a file using the Matlab interface for offline analysis.

## Method validation

Before implementing the rtSE method in human studies, it is necessary to validate that the technique can accurately estimate current source amplitudes in the presence of simulated head movement. We hypothesized that phantom (“head”) position estimation using our rtSE technique would be as accurate as a comparable offline approach. For the current sources within the phantom that simulate neural activity, we hypothesized that the real-time technique would localize the known phantom sources as accurately as the vendor-supplied off-line method. Further, we hypothesized that, while the phantom was moving, rtSE would accurately estimate the movement profile and the magnitude of the current source.

### Statistical analysis

In offline analysis, the peak-to-peak magnitude of the rtSE data obtained during current source activation was calculated using an automated local extrema detection algorithm. For each position in which the phantom was stationary, the magnitudes for all activations of the current source were averaged. This provided an estimate of the mean source amplitude at that phantom position. The percent change of these values from the mean source amplitude at the start of the scan was also calculated.

Also in offline analysis, the HPI coil positions for each second of data were obtained using the vendor-supplied MaxFilter software. This allowed for direct comparison to the HPI coil positions estimated in real-time. Displacements of the HPI coils from second to second were also calculated based on the offline algorithm to estimate the phantom velocity over time. Additionally, offline movement compensation of MEG data based on SSS [16] was also applied to the data from Experiment 3. Offline source estimation of the movement corrected MEG data was performed using the vendor-supplied software, and peak-to-peak magnitudes of current source activation were calculated as described above for the rtSE data. This provided comparative data for the accuracy of movement compensation achieved by the rtSE method.

#### Experiment #1

To address the hypothesis that real-time estimation of stationary HPI positions would be as accurate as the vendor-supplied method implemented offline, a univariate analysis of variance (ANOVA) was applied to the real-time and offline calculated coil positions. Using all 326 data segments, main effects were tested for method (real-time/offline), coil number and dimension (*α* = 0.05). All positions over time were calculated with respect to the mean position reported by the vendor-supplied method. This eliminated the irrelevant main effect due to the difference in the position of each coil on the phantom.

#### Experiment #2

To determine the localization accuracy of rtSE dipole fitting, the known location was subtracted from the estimated location for each of the eight sources. Using the calculated differences, a one-sample *t*-test was used to test for a difference from 0. This statistical test was designed to be sensitive to a significant difference from 0 in each spatial dimension (test value = 0, *α* = 0.1). A liberal alpha value of 0.1 was employed in order to confidently accept the null hypothesis that the estimated source locations using rtSE would be similar to the known source locations (i.e., with *α* = 0.1 there is a greater probability of a significant finding, thus a non-significant finding indicates a more robust similarity).

#### Experiment #3

To establish the accuracy of monitoring a moving phantom in real time, the phantom translations and rotations calculated in real-time were compared to the offline vendor-supplied results. We determined the number of data segments in which the two methods reported a greater than 2 mm or 2° disparity between real-time and vendor supplied methods. These data were subsequently categorized according to which segments also corresponded to large movements based on displacement and velocity. Results are presented as percentage of samples with large error, and percentages of samples corresponding to large values of the mentioned factors (i.e., velocity and displacement).

To investigate the accuracy of rtSE during movement, we calculated the linear regression between mean displacement of HPI coils from the original position and percent change in mean current source amplitude for the rtSE data. To determine the effectiveness of rtSE, this process was repeated for the data without movement compensation, and for data calculated using offline movement compensation and source estimation. Slope values for each of the three linear regressions were compared to test for differences between real-time and no movement compensation conditions, as well as between real-time and offline analysis conditions [17].

### Results

Statistical analysis of data from Experiment 1 revealed a significant difference between the real-time approach and vendor-supplied offline methods for the localization of stationary HPI coils (*p* < 0.05). While the real-time method for estimating head position does not provide the same value as the offline method, the magnitude of the mean difference between the methods is less than 1 mm in each cardinal axis (*x*, *y*, *z* = 0.003, 0.5, and −0.6 mm, respectively). Additionally, the standard deviations for the real-time coil position estimates across the stationary phantom scan are smaller than 1 mm (*x*, *y*, *z* = 0.4, 0.8 and 0.4 mm, respectively). This suggests that real-time localization of the HPI coils provides consistent values over the course of the scan, with some sub-millimeter, systematic difference between the real-time and offline algorithms.

The systematic difference between the real-time and offline HPI coil localization results are likely due to either excluding magnetometers, using a rigid body head position optimization, or excluding SSS analysis from the real-time algorithm. However, the differences in estimated head position are small, in particular when compared to the normal range of head movement experienced during functional neuroimaging (i.e., ∼1–10 mm). For many users, the small differences noted between real-time and offline HPI localization will represent an acceptable trade-off to allow for the implementation of real-time applications in MEG.

In Experiment 2, real-time averaging of repetitive current source activation and ECD fitting accurately localized the eight phantom sources. Mean differences between the measured and known positions across all dipoles were less than 1 mm (*x*, *y*, *z* = 0.002, 0.03, 0.1 mm respectively). No significant differences (*p* = 0.1) were found in any spatial dimension between the real-time estimated positions and the known locations.

**Fig. 1:**
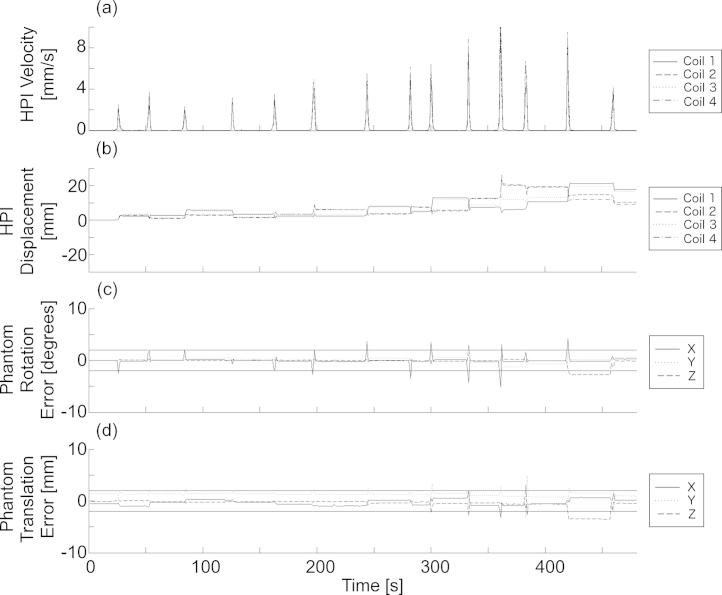
The accuracy of real-time head position estimation over time is shown. The velocity [mm/s] (a) and displacement [mm] (b) of the four HPI coils, as calculated using the vendor-supplied offline method is shown. As well, the difference between real-time and offline methods for calculating translation (c) and rotation (d) of the phantom over time are shown. Solid horizontal lines on (c) and (d) indicate a threshold of 2 mm or 2°. Differences between real-time and offline methods larger than the threshold occur only during large movement and following large displacement from the original position.

The accuracy of real-time head position estimation during movement of the phantom (Experiment 3) is displayed in Fig. 1. Real-time and offline vendor-supplied estimates of phantom translation and rotation agreed to within 2 mm or 2° for 88% of data segments. The samples that are associated with differences in estimated rotation or translation correspond to data segments when the phantom was moving or when the displacement of the phantom was large (1.7 cm from the original position).

**Fig. 2:**
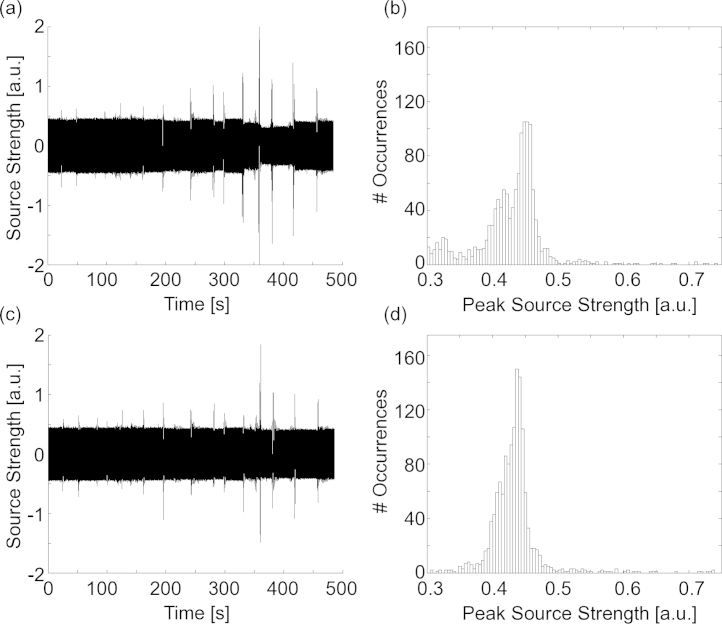
Source amplitudes over time with (a) and without (c) rtSE movement compensation are shown. The estimated peak magnitudes for each activation of the calibrated source are shown as histograms for data analysed with (b) and without (d) rtSE movement compensation.

Fig. 2 shows the source estimation of a segment of data with an active current source (Experiment 3). The source estimation is shown with and without real-time movement compensation applied. During actual movement, there is a large increase in signal amplitude, likely due to poor estimation of magnetic field patterns over the 1 s interval. After this, the time courses stabilize for both methods. Qualitatively, one can see that, following movement, the rtSE source amplitude returns to a value closer to the original amplitude (time zero) than source estimation without movement compensation. Further, source amplitudes appear to be underestimated without rtSE. This effect is clear in the accompanying histograms of estimated peak source strength, which show a shift toward lower estimated source strength.

**Fig. 3:**
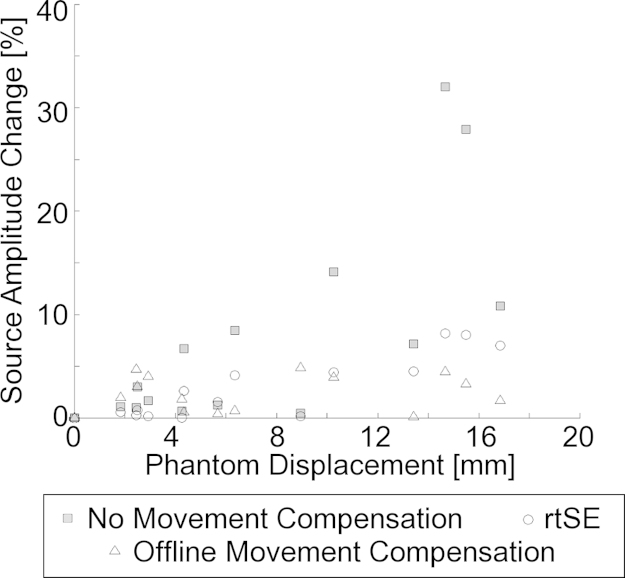
Accuracy of source estimation is shown as a function of HPI displacement. Mean percent change in source amplitude, evaluated at each static position of the phantom, is shown for offline movement compensation followed by source estimation calculation, source estimation calculation with no movement compensation, and rtSE. For displacements less than 10 mm, rtSE is as accurate at estimating source strength as the vendor supplied offline method.

Fig. 3 shows the percent change in mean current source amplitude from baseline as a function of the displacement of the HPI coils from initial positions. Data are plotted for real-time, offline, and no movement compensation. Slopes for the linearly regressed datasets are 0.48 ± 0.07%/mm (rtSE), 0.05 ± 0.09%/mm (offline movement compensation) and 1.4 ± 0.3%/mm (no movement compensation). Comparison of the slope values across conditions showed significant differences between rtSE with and without movement compensation (*p* < 0.05). Also, a significant difference was found between the slope values for the rtSE and offline methods (*p* < 0.001). These results suggest that the rtSE method improves the accuracy of current source estimation when the phantom is displaced from the original position. However, rtSE is not as accurate as the offline source estimation approach, particularly for movements greater than 10 mm.

## Additional information

Functional neuroimaging data provide an estimate of where and when activity occurs in the brain. Real-time analysis of this neuroimaging data allows for the participant's measured brain signals to be incorporated into the experimental design. These designs include brain–computer interface (BCI) [18–20,21] or the provision of neurofeedback. In the latter application, the user receives a measure of their own brain activity in real-time, often for the purposes of neurorehabilitation [22–24].

Magnetoencephalography (MEG) is one neuroimaging technique that can be used for such applications, providing millisecond temporal resolution and millimeter source localization accuracy [25–27]. In MEG, sources are localized in the coordinate frame of the MEG device. A conversion to the coordinate frame of the participant requires the accurate tracking of head position within the scanner [1]. To calculate head position, HPI coils, placed at common anatomical landmarks on the head, are localized during the scan, generating a continuous estimate of head position. Monitoring of head position combined with source-level analysis provides the capacity to localize brain activity even when significant head movement occurs.

As an alternative to existing approaches to source estimation with head movement compensation, we present a real-time source estimation (rtSE) method that corrects for head movement in order to maintain accurate estimates of source-level brain activity when the head is moving. In the current study, the rtSE method is validated by simulating head movement using a phantom with known source strength. The rtSE method completes the analysis on a sub-second time scale, providing a foundation for future work permitting the use of the resultant source-level data for the provision of neurofeedback or use in a BCI application. Neurofeedback- and BCI-based applications are increasingly being used as therapeutic interventions in rehabilitation [28,29]. Importantly, the rtSE method acts as a computationally inexpensive alternative that opens the door to neurofeedback applications in populations that may have difficulty remaining still during functional neuroimaging (e.g., patients, children).
